# Old cogs, new clocks: a conserved protein complex controls developmental and circadian timing

**DOI:** 10.1038/s44318-025-00586-y

**Published:** 2025-10-20

**Authors:** Ka Yi Hui, Jürgen A Ripperger

**Affiliations:** Department of Biology/Biochemistry, University of Fribourg, 1700 Fribourg, Switzerland

**Keywords:** Development, Signal Transduction

## Abstract

A recent study identifies a conserved timing module composed of LIN-42/PER and KIN-20/CK1that is shared by circadian and developmental timing programs.

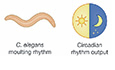

## Teaser

The circadian clock protein Period and its *C. elegans* counterpart LIN-42, involved in regulation of daily rhythms and developmental timing, respectively, have long been suspected to function mechanistically in a similar manner. The new study by Spangler et al, (2025) in The EMBO Journal reveals that LIN-42 forms a functional complex with the CkIδ/ε kinase homologue KIN-20, establishing the first conserved timing module shared between circadian and developmental timing programs.

Biological timing operates at various timeframes, from the ~24-h circadian rhythms that anticipate daily environmental changes to the precisely choreographed sequences of developmental events. These timing systems appear fundamentally different, one cyclical and recurring, the other mainly linear and progressive. Despite this, they share a surprising number of molecular components. The mammalian circadian clock relies heavily on the Period (Per) proteins, which interact with casein kinase 1 δ/ε (CK1δ/ε) to generate the delays necessary for ~24-h rhythms (Lowrey et al, 2000; Takahashi, 2017). In the nematode *Caenorhabditis elegans*, the Per orthologue LIN-42 controls developmental timing rather than circadian rhythms, regulating both the sequence of developmental events (heterochrony) and the rhythmic occurrence of moulting (Jeon et al, 1999).

It is unclear how similar and conserved the molecular mechanism between these two homologous proteins is in different timing systems. The answer to this question will provide insight into how different timing mechanisms evolved by repurposing basic timing molecular components across various biological contexts and timeframes.

To understand the LIN-42 timing machine essential for moulting, Spangler, Braun, Ashley, and colleagues systematically dissected LIN-42’s functional domains using precise genetic deletions in *C. elegans*. Their findings overturn long-held assumptions about which parts of LIN-42 are critical for its timing functions. Surprisingly, deletion of the PAS domains, the most conserved feature between LIN-42 and mammalian Per proteins, caused only mild developmental defects. Instead, the authors discovered that two previously understudied sequence motifs, termed SYQ and LT, are essential for rhythmic moulting.

Through elegant biochemical experiments, the team demonstrated that these SYQ and LT motifs constitute a functional CK1-binding domain (CK1BD) that anchors KIN-20, the *C. elegans* CK1 orthologue. Animals lacking either the LIN-42 CK1BD or KIN-20 kinase activity exhibited severely disrupted moulting rhythms, with individual animals showing various lengths of moulting times, in contrast to the synchronised moulting timing observed in a normal population.

The parallels between the LIN-42-KIN-20 complex and its mammalian Per-CK1δ/ε counterpart extend beyond simple protein-protein interactions. Like mammalian Per, LIN-42 undergoes extensive phosphorylation by its associated kinase, and this phosphorylation exhibits the same pattern of feedback inhibition seen in circadian clocks. The two CK1BD subdomains play distinct roles: one primarily anchors the kinase for stable binding, and the other modulates kinase activity and product release, similar to the mammalian system (Philpott et al, 2023).

Interestingly, the authors found that LIN-42 does not simply serve as a substrate for KIN-20 but actively regulates the kinase’s cellular localisation. KIN-20 exhibits dynamic shuttling between the nucleus and cytoplasm that coincides with the developmental stage, which depends on its interaction with LIN-42. Loss of either the LIN-42 CK1BD or its phosphorylated C-terminal tail reduces KIN-20’s nuclear accumulation, suggesting that LIN-42 serves as both a substrate and a regulator of its associated kinase.

**Figure 1:**
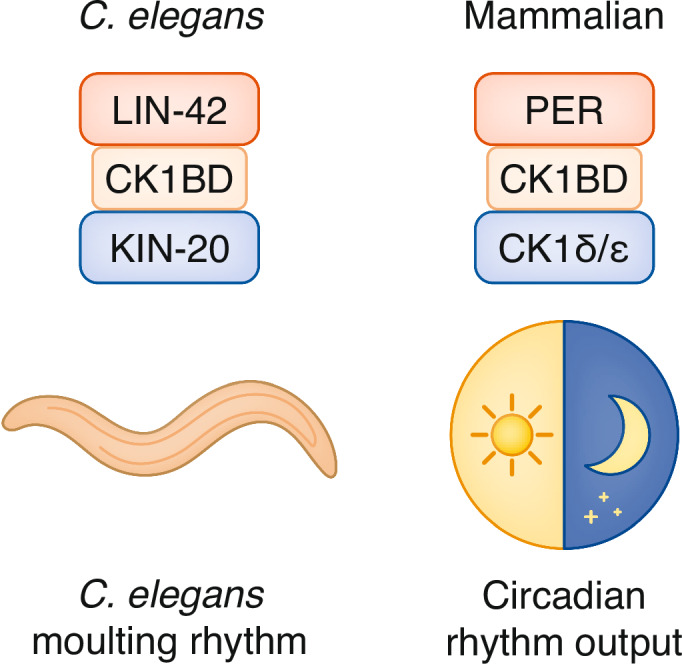
A schematic illustration showing the conserved LIN-42-KIN-20 and Per-CK1δ/ε complexes side by side. For the *C. elegans* system (left): LIN-42 protein with its CK1BD region binds to KIN-20, with the moulting rhythm as output. For the mammalian system (right): Per protein, with its CK1BD interacts with CK1δ/ε, leading to a circadian rhythm output.

These findings establish the LIN-42-KIN-20 complex as the first example of a functionally conserved timing module shared between circadian and developmental timing systems (Fig. 1). This conservation suggests that certain regulatory mechanisms are so fundamental to biological timing that they have been maintained across vast evolutionary distances and co-opted for different temporal contexts.

The work also provides new insights into how Per-CK1δ/ε complexes might function in mammalian circadian clocks. While much attention has focused on how CK1δ/ε regulates Per stability and hence delays nuclear entry, the *C. elegans* results highlight the potential reciprocal regulation of CK1δ/ε by Per. This bidirectional control may be crucial for the precision and robustness of biological timing systems (Aryal et al, 2017).

Located in the neurons of adult *C. elegans*, the complex of LIN-42 and KIN-20 may regulate rhythmic events on a more daily basis (Lamberti et al, 2024). It would be interesting to compare the modes of interaction between the complex involved in moulting to the one in adult *C. elegans*. It was also demonstrated that LIN-42 could interact with the nuclear receptor NHR-85, which is also involved in the regulation of moulting (Kinney et al, 2023). Essentially the same interaction was found between the nuclear receptor REV-ERBα and PERIOD2 in the mouse circadian oscillator (Schmutz et al, 2010), suggesting another parallel between the worm and the mammalian system.

The identification of conserved timing modules raises fascinating questions about the evolutionary relationship between different timing systems. Did developmental timing mechanisms give rise to circadian clocks, or vice versa? The evidence from *C. elegans* suggests that LIN-42’s role in rhythmic moulting, which involves recurring cycles of activity, may represent an evolutionary intermediate between purely sequential developmental programs and fully autonomous circadian oscillators.

Interestingly, tracing back the evolution of bacteria to about 1 billion years ago, the circadian oscillator of cyanobacteria may not have been capable of providing self-sustaining rhythms, even if the key components that can perform this task today were already in place (Mukaiyama et al, 2025). Hence, even for these simpler organisms, self-sustaining circadian rhythms may have evolved from a simpler timing mechanism over many generations.

From a practical standpoint, understanding these conserved mechanisms could inform therapeutic approaches for circadian rhythm disorders. If the fundamental regulatory logic of Per-CK1δ/ε complexes is conserved across timing systems, insights from developmental biology might reveal new strategies for modulating circadian function.

The work by Spangler, Brown, Ashley and colleagues also highlights the power of combining precise genetic tools with biochemical approaches to dissect complex biological systems. Their systematic domain analysis revealed unexpected functional relationships that might have been missed by studying whole-protein knockouts or overexpression systems alone.

As we continue to uncover the molecular basis of biological timing, it is becoming clear that the precise timing of events represents one of biology’s most fundamental and conserved regulatory mechanisms. The LIN-42-KIN-20 complex serves as an interesting example of how a simple timing mechanism is adapted for different biological contexts.
